# Explicit-memory multiresolution adaptive framework for speech and music separation

**DOI:** 10.1186/s13636-023-00286-7

**Published:** 2023-05-09

**Authors:** Ashwin Bellur, Karan Thakkar, Mounya Elhilali

**Affiliations:** Electrical and Computer Engineering, Johns Hopkins University, Baltimore, USA

**Keywords:** Auditory system, Speech enhancement, Music separation, Multi-scale redundant representations, Temporal coherence, Explicit memory

## Abstract

The human auditory system employs a number of principles to facilitate the selection of perceptually separated streams from a complex sound mixture. The brain leverages multi-scale redundant representations of the input and uses memory (or priors) to guide the selection of a target sound from the input mixture. Moreover, feedback mechanisms refine the memory constructs resulting in further improvement of selectivity of a particular sound object amidst dynamic backgrounds. The present study proposes a unified end-to-end computational framework that mimics these principles for sound source separation applied to both speech and music mixtures. While the problems of speech enhancement and music separation have often been tackled separately due to constraints and specificities of each signal domain, the current work posits that common principles for sound source separation are domain-agnostic. In the proposed scheme, parallel and hierarchical convolutional paths map input mixtures onto redundant but distributed higher-dimensional subspaces and utilize the concept of temporal coherence to gate the selection of embeddings belonging to a target stream abstracted in memory. These explicit memories are further refined through self-feedback from incoming observations in order to improve the system’s selectivity when faced with unknown backgrounds. The model yields stable outcomes of source separation for both speech and music mixtures and demonstrates benefits of explicit memory as a powerful representation of priors that guide information selection from complex inputs.

## Introduction

The human brain solves complex auditory tasks such as having a conversation in a busy cafe or picking the melodic lines of a particular instrument in an ensemble orchestra. While seemingly effortless, these tasks are a real feat given that the brain only has access to a low dimensional pressure waveform of the mixture as the primary signal and uses a common front-end pipeline to process incoming signals, regardless of their complexity [1]. This sound mixture composed of different sources is analyzed through common processing stages in the auditory system to separate it into perceptual auditory objects of unequal cognitive value. Some streams of interest are promoted to the foreground, while others are relegated to the background, e.g., attending to the voice of a friend and ignoring the background chatter in a cafeteria or jamming to beats of the drum and ignoring the melody of the accompaniments. Critical to this separation process is the mechanism of attention which gates the selection of foreground objects [2, 3], hence allowing the system to focus its computational resources on signals of interest. Priors stored in memory are deployed as needed and used to narrow down the representational space of targets of interest to the system [4].

Neuroscience research has shed light on some of the mechanisms and neuronal architectures that facilitate adaptive listening [5–9]. A series of transformations along the auditory pathway map the low dimensional pressure waveform to a higher dimensional space [10–12]. Studies have shown that naturally occurring sound objects have distinguishable characteristics and occupy non-overlapping regions in this high dimensional space, enabling the grouping of these features into perceptual auditory objects [13, 14]. In addition, functional magnetic resonance imaging (fMRI) findings have indicated the presence of a spatially distributed network architecture in cortical regions with localized areas capturing different parts of the high dimensional spectrotemporal modulation space [15, 16]. Further, selective attention gates the representation of incoming signals that are temporally coherent with attended priors [17]. In addition, there is evidence that memory priors guiding selection of attended inputs function in a distributed setup rather than a unitary system, complementing the distributed feature encoding process [18–21]. These priors themselves undergo continuous adaptation and sharpening as a result of selective attention [22, 23], likely allowing the system to adapt to novel contexts and changing interfering backgrounds.

Inspired by these principles, numerous efforts in machine hearing have leveraged these principles to process audio signals. Hierarchical and multi-resolution schemes have been widely effective in providing rich and redundant mappings of sound inputs, particularly for the task of source separation. Grais and colleagues proposed a multi-resolution architecture using convolutional filters of varying sizes to capture different parts of the spectrotemporal modulation space for source separation [24, 25]. Hierarchical U-networks and residual networks based on skip connections have also resulted in compelling performance leaps for tasks such as singing voice separation [26] and music source separation [27, 28] by exploring features at various levels of abstraction. Most systems adopting a multi-resolution framework generally operate within a single end-to-end network, hence resulting in highly effective, yet very specialized systems, optimized for either speech inputs, music signals, or other sound events. These approaches raise the question regarding the effectiveness and commonality of principles that facilitate segregation of sounds regardless of sound class, be it speech, music, or other audio signals. After all, human brains are generalist systems able to process various sound inputs and attend to different objects of interest.

Beyond multi-resolution architectures, attention has been widely used as a mechanism that further guides processing in deep networks by incorporating weighting of local and global contexts, as shown by recent performance leaps of models such as transformers and conformers [29]. Throughout the use of attentional processes in deep learning, the term attention takes on a meaning of a soft search across the feature space whether it is in the form of self-attention or weighting of contexts. In the present work, attention refers to a more biological construct, namely as a gating operation that is guided by explicit memory or priors to change the output of the system to the same input to attend to sound A or sound B in the mixture. These explicit memories act as information bottlenecks that refine the network during inference and operate as discrete and compact units that guide the processing of the entire system.

**Fig. 1:**
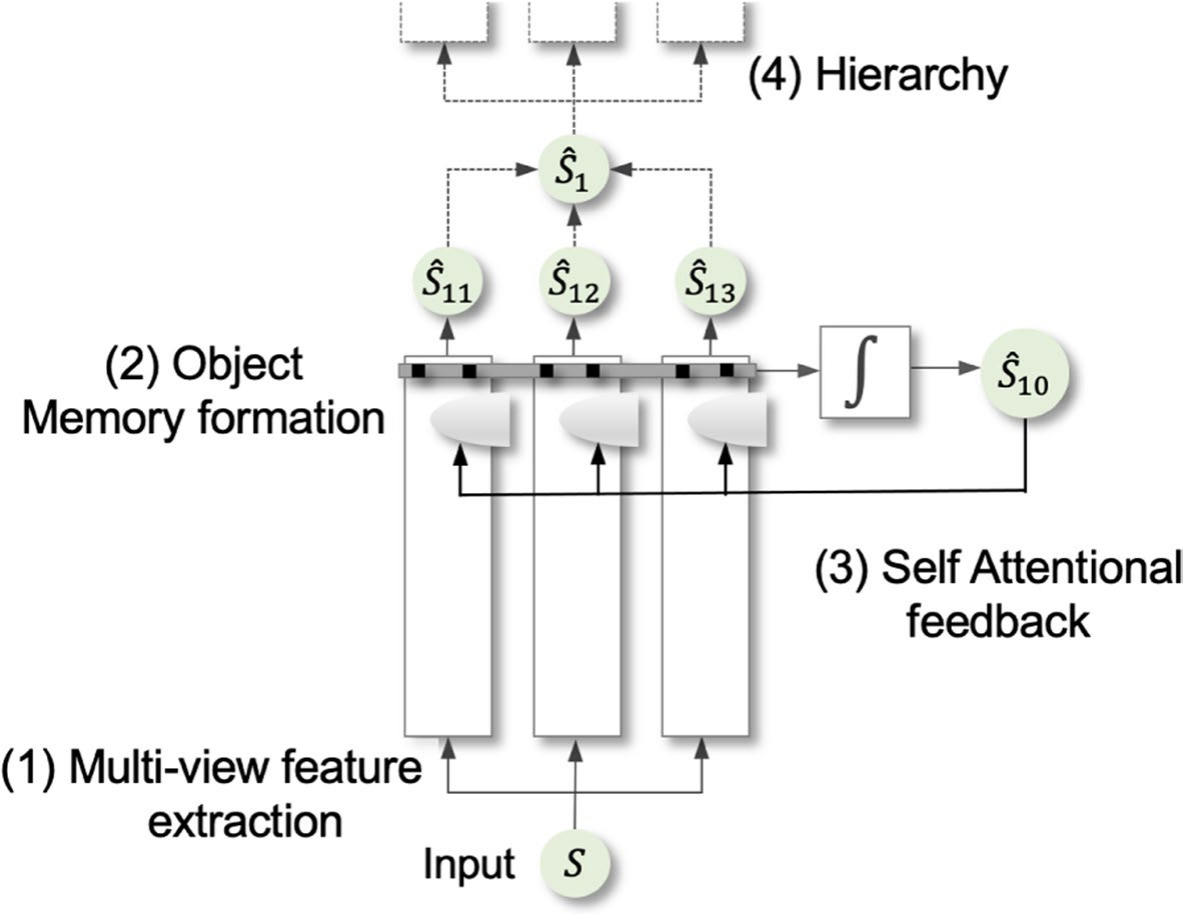
A brief view of the proposed framework incorporating the following bio-inspired design elements: (1) multi-view feature extraction with parallel streams trained independently to yield multiple read-outs, (2) a distributed network of memories for targets of interest used at each local stream to gate embeddings of interest, (3) feedback from the integrated output is propagated to re-tune local memories during inference (specialist system), (4) the architecture can be extended across multiple levels of a hierarchy

In the present study, we propose a framework that achieves a dual objective in the context of audio source separation: (i) a universal, modular framework that operates on both speech and music signals by forming explicit object memories and (ii) an adaptive system that selectively re-tunes memory to adapt to changes in the soundscape. The proposed system represented in Fig. 1 incorporates four key bio-mimetic principles:

1. *Multi-view feature extraction*: Multiple parameterized independent streams trained in parallel to capture information from different vantage points [15, 16]. In addition, a stream integrator is trained to combine views across parallel streams.
2. *Object memory formation*: An explicit memory of different auditory objects is learned to represent patterns of a particular object captured across multiple views [30, 31]. These memories are used to gate features for target auditory objects.
3. *Self-attentional feedback*: During inference, self-attentional feedback allows memory representations in each stream to refine themselves by using information from a different vantage point in the network, in order to enhance perceptual fidelity when faced with changing backgrounds [22].
4. *Hierarchy*: The basic architecture can be repeated across different hierarchical levels. Self-feedback not only modulates parallel streams within a layer but trickles down to earlier levels in the hierarchy.

We evaluate this model for two different tasks, without any adjustment to design elements or system architecture. In other words, the same model architecture is trained to attend to target speech in presence of various noise distortions (speech enhancement) and trained to attend to a particular instrument in presence of different instrumentals or vocals (music source separation). Both models differ in the number of possible targets, i.e., 2 for speech enhancement (speech and noise) and 4 for music separation (bass, drums, vocals, and other). It is important to note that the proposed scheme frames the source separation problem as one where the system is attending to one auditory target (based on priors in the memory network) and ignores all others. As such, the network only outputs the foreground signal desired to be listened to (as indicated by the user), rather than multiple signals in an input mixture.

The analysis to follow evaluates the effectiveness of the above mentioned mechanisms in an agnostic source separation task without exclusively targeting computational cost or high-performance computing to outperform state-of-the-art systems. In Section 2, we describe the proposed framework in detail; followed by the training process, datasets employed and baseline systems in Section 3. The results are presented in Section 4, while Section 5 discusses the capacity and constraints of the proposed system in mimicking the biological system.

## Explicit-memory multiresolution adaptive (EMMA) framework

**Fig. 2:**
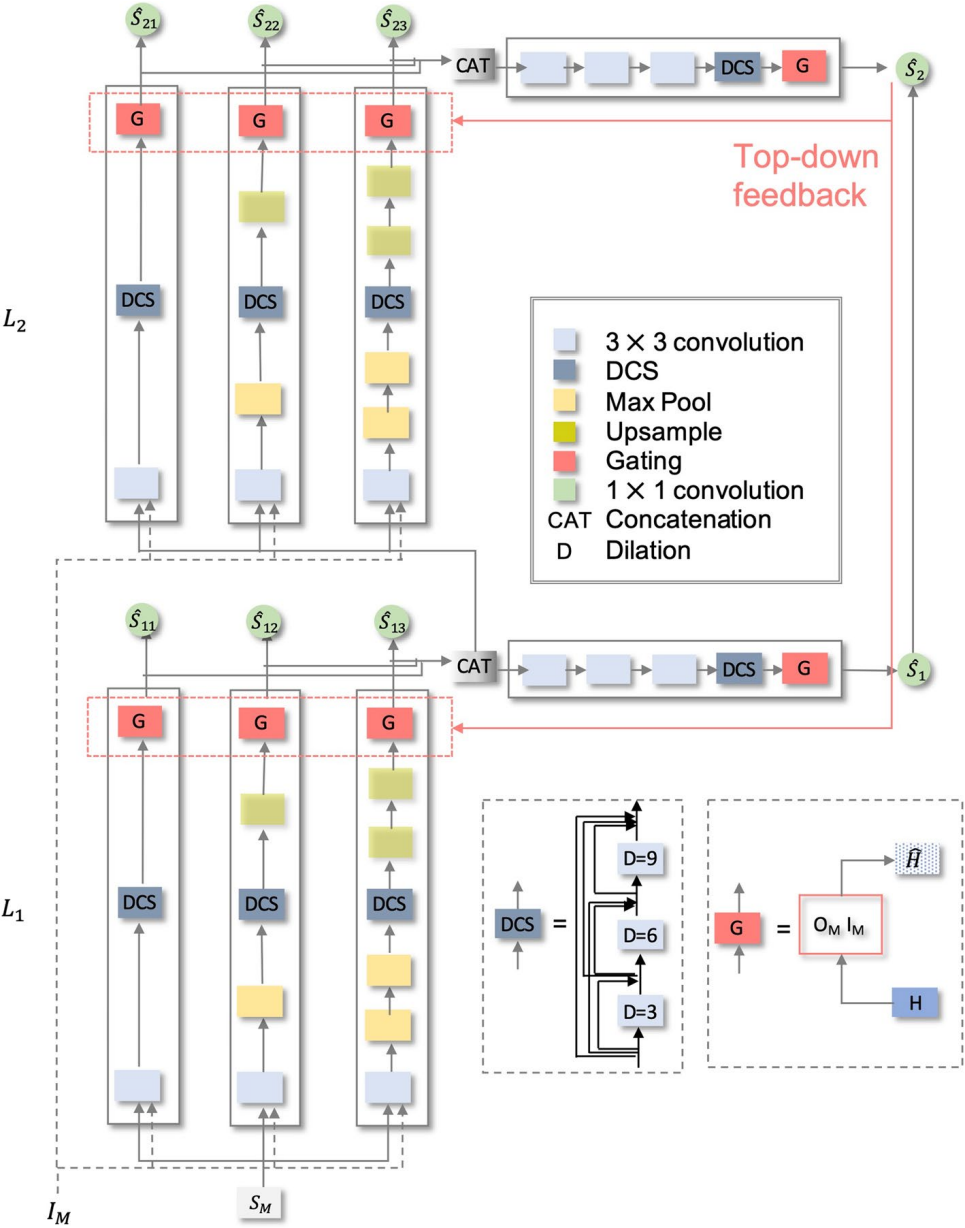
Detailed architecture of the proposed explicit memory multiresolution adaptive (EMMA) framework

Expanding upon the goals described in Fig. 1, we propose the explicit memory multi-resolution adaptive (EMMA) framework as represented in Fig. 2. The model takes as input two quantities, the magnitude short-time Fourier transform (STFT) of the mixture signal denoted by $S_M$ and an indicator variable $I_M \in \mathbb {N}^O$ representing the target object. *O* denotes the maximum number of objects that can be present in the system and is pre-defined for the task. $O=4$ for music source separation and represents memories of vocals, bass, drums, and others, while $O=2$ for speech enhancement and represents memories of speech and background distractors. The desired output from each of the parallel streams as well as stream integrator is the magnitude STFT of the attended auditory object *only* i.e., $o \in \{1,2,... O\}$.

The proposed model has four key components: (1) different levels of the hierarchy, each composed of parallel processing streams; (2) a stream integrator at each level of the hierarchy that renders a unified view across streams; (3) memory informed attentional gating *G* operating at end of each stream, and (4) self-attentional feedback to re-tune memories during inference. The following subsections describe each component in detail.

### Parallel multi-resolution feature analysis

As described previously, the network consists of parallel streams, each trained independently. Let the $j^{th}$ stream output of the $i^{th}$ layer be represented by $\hat{S}_{i,j}$, where $i \in \{1, 2, ... i_{max}\}$ and $j \in \{0, 1, 2, ..., j_{max-1}\}$. We formulate the prediction of a stream $f_{\theta _{i,j}}$ parameterized by $\theta _{i,j}$ as follows:-

1 $$\begin{aligned} \hat{S}_{ij}=\left\{ \begin{array}{ll} f_{\theta _{i,j}}(S_{m}, I_m) \quad \text {if } i = 1 &{} {}\\ f_{\theta _{i,j}}([\hat{S}_{i-1,1}| \hat{S}_{i-1,2}| \ldots | \hat{S}_{i-1,j_{max-1}}] ,I_{m}) &{} \text {else} \end{array}\right. \end{aligned}$$

where $[\hat{S}_{i-1,1}| \hat{S}_{i-1,2}| \ldots | \hat{S}_{i-1,j_{max}-1}]$ represents the concatenated outputs of the previous layer’s streams except the stream integrator. Here, $i_{max}$ and $j_{max}$ denote the total number of layers and count of streams per layer respectively; $j=0$ represents the stream integrator. In our experiments, we fix $i_{max}=2$ and $j_{max}=4$. The input of each of the streams in level 1 is the magnitude STFT of the mixture $S_M$; while the input of each of the streams in level 2 is the concatenated output embeddings from the independent streams of level 1. All streams also take as input an indicator variable $I_M \in \mathbb {N}^O$, which signifies the target object in the scene.

Each stream consists of a two-dimensional convolution node as the basic computational unit, followed by a max pooling operation for some paths. As illustrated in Fig.2, the streams differ in the number of pooling operations and upsampling blocks attributing to $S_{11}$ and $S_{21}$ being the fastest, $S_{12}$ and $S_{22}$ as the medium level and $S_{13}$ and $S_{23}$ capturing the slowest modulation features. All streams use a dilated-convolution stack (DCS) (Fig. 2) which is used to enable estimating filters of varying resolutions, with the most dilated filters capturing the slower scale features [32]. The final stage in a stream is the gating operator *G* that is used to attend to the object of interest and is described later in this section.

### Stream integration

Individual streams capture the acoustic scene and its constituent objects at differing levels of resolution and abstraction depending on the parameters of the stream and its position in the hierarchy. We hypothesize that these streams capture the acoustic scene from different vantage points, and integrating information across streams in different hierarchical levels should lead to better target separation. Effectively, stream integrators at each level act as read-outs of the auditory objects from a particular level and have similar architectures as each of the parallel streams. They consist of convolutional nodes and DCS, albeit deeper in terms of network complexity. The input to stream integrators is the concatenated embeddings of streams from the corresponding level of hierarchy along with indicator variables $I_M$. The output of a stream integerator is formulated as follows

2 $$\begin{aligned} \hat{S}_{i,0}=f_{\theta _{i,0}}([\hat{S}_{i,1}| \hat{S}_{i,2}| \ldots | \hat{S}_{i,j_{max}-1}] ,I_{m}) \end{aligned}$$

### Memory informed attentional gating

The attentional gating G is inspired by the principle of temporal coherence which states that when attention is directed towards a characteristic feature of the target object, this characteristic feature acts as an anchor, and all the features that are coherent with temporal activations of this anchor become bound together to form a common auditory object [17, 33]. In other words, the attentional mechanism leverages patterns in the memory to weight embeddings that are in sync with the memory of interest for further processing [34].

**Fig. 3:**
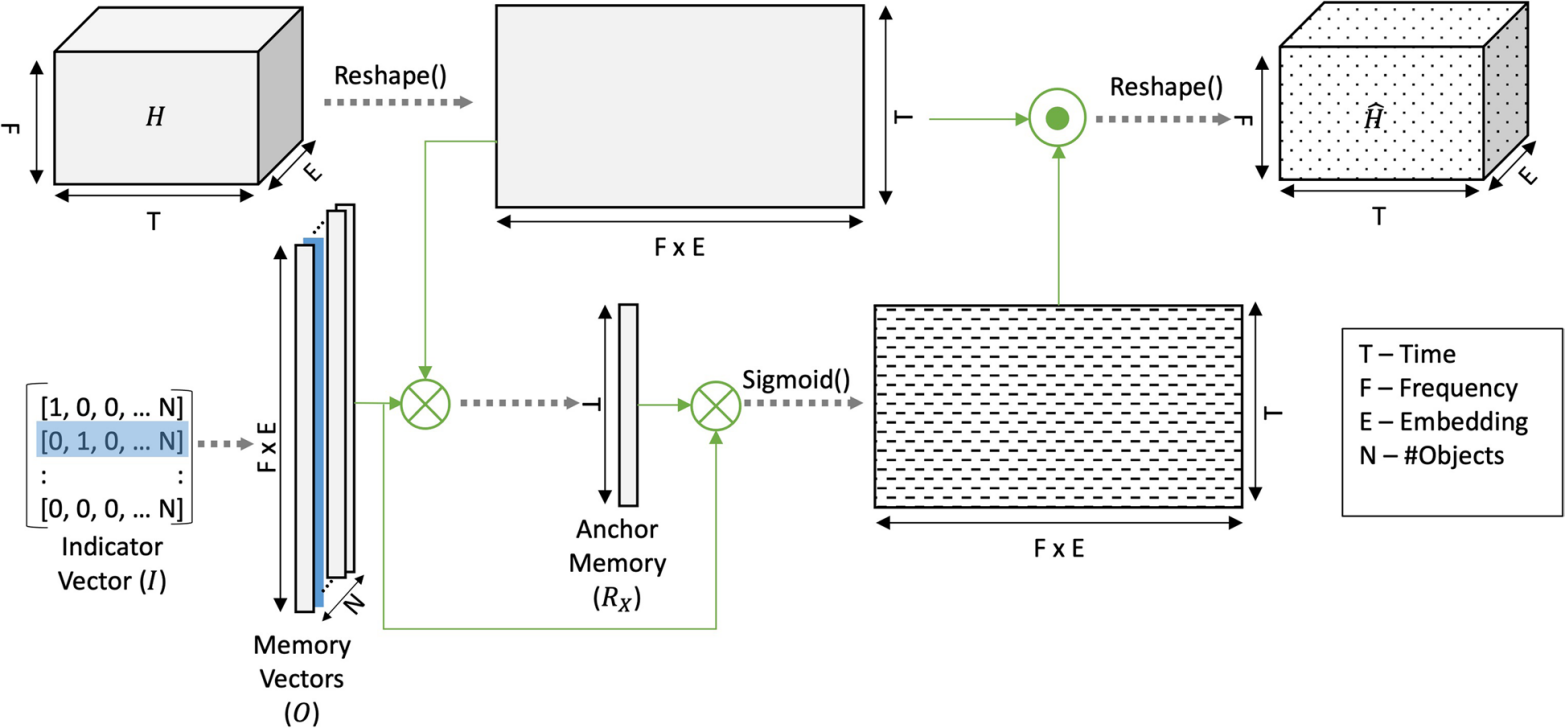
Visual of the proposed memory informed attentional gating for a single sample. The attention module takes a single batch of representations (*H*) as the input and reshapes it to (*q*, (*p*.*r*)). Utilizing reshaped $\tilde{H}$ and the selected object memory vector $O_x$, we estimate the anchor memory $R_x$. In the next step, we gate the embeddings that do not fire coherently with the Anchor Memory and produce the gated representation $\hat{H}$

Let *H* signify the embeddings of the mixture in a 3-D space with dimensions $p \times q \times r$. The dimensions *p*, *q*, and *r* represent frequency channels, number of time frames, and number of hidden units respectively. For simplicity, assume we want to segregate the audio into 2 sound objects, object A and object B. Let $O_M$ denote the object memories of dimensions $p \times r \times 2$ and $I_M$ of dimensions $2 \times 1$ be the indicator variable indicating the object we are interested in extracting from the audio. The gated attentional block performs the following operations (Fig. 3):

1. First, the memory vector $O_{x}$ is selected from the vector $O_{M}$ as guided by the indicator $I_{M}$.
2. Next, the weighted activation pattern $R_x$ (also referred to as anchor memory) is estimated from the memory $O_{x}$ and reshaped mixture embedding $\tilde{H}$ using the following equation.
  3 $$\begin{aligned} R_x[1,t] = \sum _{i=1}^p \sum _{j=1}^rH[i,t,j]*O_x[i,j,1] \; \forall t \in \{1,...,q\} \end{aligned}$$
3. The anchored memory is then matrix multiplied with the memory vector to mimic the object memory’s pattern along the time dimension.
  4 $$\begin{aligned} \hat{R}_{x} = O_x*R_x \end{aligned}$$
4. Resultant matrix $\hat{R}_{x}$ is of the same size as $\tilde{H}$ which undergoes a sigmoid non-linearity. Following the non-linearity, this matrix is then used as a gating operator (via element-wise multiplication) with the $\tilde{H}$. This step is represented mathematically in Eq. 5.
  5 $$\begin{aligned} \hat{H} = H \odot sigmoid(\hat{R}_{x}) \end{aligned}$$

This process is similar to the use of a dictionary basis to segregate sources within the non-negative matrix factorization paradigm [35]. The object memories are analogous to dictionary bases in this scenario and the gating operation is performed by reshaping the embeddings over time without non-negative constraints.

In each of the independent streams and stream integrators, source separation or enhancement is performed through the penultimate block in the stream, indicated as G. The gating block takes the output of the previous block and indicator as the input and returns the embeddings activated in coherence to the pattern learned in the memory. The local attention gating block in each of the parallel streams and stream integrators is followed by the read-out layer, a $1 \times 1$ convolution to generate the output spectrogram from each of the streams.

### Self-attentional feedback

The network proposed thus far consists of multiple streams across different levels of hierarchy with stream integrator at each level integrating information across the streams. By design, the network strives to represent distinct views of information across its different paths as information flows to integrator streams and higher levels. Each stream contains its own memory of objects used to separate sources. Deployment of top-down feedback is used to refine the memories of lower layers based on output from upper layers. This top-down process focuses on only re-tuning the streams’ object memories in the attentional gating blocks of individual streams by minimizing the objective $\mathscr{L}$ defined as,

6 $$\begin{aligned} \mathscr{L} = \sum _{i=1}^{i_{max}}\sum _{j=1}^{j_{max}} \mathscr {D} (\hat{S}_C, \hat{S}_{i,j}) \end{aligned}$$

where, $\hat{S}_C = \sum _{k=1}^{i_{max}}S_{k, 0}$ and $\mathscr {D}$ is the distance metric used to quantify the distance between $\hat{S}_C$ and $\hat{S}_{i,j}$. This is illustrated in Fig. 2, where only the object memories denoted by “G” in the red dotted boxes are updated while the rest of the distributed network is kept fixed.

## Experiments

In this section, we describe the datasets, network configuration, training, and re-tuning strategy in detail for both speech and music separation. The network configuration, training, and fine-tuning strategies are similar for both tasks, hence it is described together for both tasks in the subsection below.

### Network configuration

In our experiments, we fix the number of layers as 2 and the number of streams per level as 3, with one stream integrator for each level, but the basic concept can be extended to more streams and levels. The network takes as input the magnitude spectrogram of the sound mixture and indicator with dimensions $B \times 2048 \times 64$ and $B \times O$ respectively, where *B* denotes batch size and *O* represents the number of objects. STFT is calculated using a window size of 2048 and a hop size of 512 with a hamming window. The number of time frames in the input is fixed to 64 and with zero padding where necessary. The indicator is one hot encoded vector representing which memory to select for further processing ($O=2$ for speech and $O=4$ for music). All max-pooling operations are performed using a kernel size 2 and stride 2. Each convolution node consists of 128 hidden units, $3 \times 3$ kernel, and “same” padding with leaky ReLU (rectified linear unit) activation [36]. Dilations are used in the DCS block (Fig. 2). Finally, the segregated waveform is obtained by taking the inverse STFT using the output magnitude spectrogram and the original mixture phase.

### Training the network

Each stream including the stream integrator is trained independently while keeping the rest of the network fixed. Firstly, the three parallel streams in level 1 are trained with the magnitude spectrogram of the music or speech mixture as input along with information of the desired target object indicated by $I_M$. Next, the stream integrator in level 1 and parallel streams in level 2 are trained with the concatenated embeddings from level 1 and indicator variable $I_M$ as input. The parameters of the parallel streams in level 1 are kept fixed when training higher levels. Finally, the stream integrator in level 2 is trained using the concatenated gated embeddings of level 2 and indicator variable $I_M$ as input, keeping the rest of the already trained networks fixed. Each stream is trained using the Adam optimizer for 35,000 iterations with a learning rate of $10^{-4}$.

To train the parallel streams, let $\hat{S}_{i, j}$ denote the output of the stream *j* in level *i* and *Y* be the ground truth (clean magnitude spectrogram) of the desired (attended) object; we employ the $L_{1}$ norm as the loss function to train each of the streams, following Eq. 7. The stream integrator network in level 1 is also trained using the $L_{1}$ norm (Eq. 8), while the stream integrator network in level 2 follows a modified loss equation (Eq. 9). This modified loss function balances a reconstruction of the clean spectrogram of the desired auditory object while enhancing contrastive information captured by the stream integrator in level 1. The parameter $\alpha \in [0,1]$ is used to balance the priorities between reconstruction and extracting complementary information. In this study, $\alpha$ is set to 0.2 following empirical investigations. The training procedure follows a sequential order in which the sample order is presented in an orderly fashion for each memory, hence maintaining a similar context. This structure yields better performance relative to random sampling, likely because the gradients calculated in the sequential strategy guarantee an equal number of updates for all object memories.

7 $$\begin{aligned} \mathscr {L}_{||}&=||Y-\hat{S}_{i, j}||_{1,1} \end{aligned}$$

8 $$\begin{aligned} \mathscr {L}_{int1}&=||Y-\hat{S}_{1, 0}||_{1,1} \end{aligned}$$

9 $$\begin{aligned} \mathscr {L}_{int2}&=|Y-\hat{S}_{2, 0}||_{1,1} - \alpha ||\hat{S}_{1,0} -\hat{S}_{2,0}||_{1,1} \end{aligned}$$

### Re-tuning the network

The re-tuning procedure uses the loss function presented in Eq.6, where $\hat{S}_C$ represents the sum of stream integrator outputs at all levels and *D* is the L1 distance between $\hat{S}_C$ and $\hat{S}_{i,j}$, $i_{max}=2$ and $j_{max}=3$. This stage is a true feedback operation that uses no ground truth data. Instead, it allows the model to leverage information from a better viewpoint to improve itself. This mechanism can also be attributed loosely to a self-correcting mechanism of the proposed EMMA framework. For the network re-tuning, the learning rate is set to 10e−5, and the gradients of the memory matrix are updated for 10 epochs at max with early stopping applied to avoid overfitting.

### Experimental tasks and baseline systems

#### Music source separation (MSS)

For this task, the MUSDB18 dataset [37] is utilized. The dataset consists of 150 songs (10 h of audio) divided into 100 train and 50 test full-track songs. The audio is provided in a multitrack 22 kHz format composed of 5 stereo streams, with the 5 streams corresponding to the *mixture, drums, bass, vocals*, and *other* instruments. For each file, the mixture corresponds to the sum of all the signals. All tracks are downsampled to 16 kHz before calculating the STFT. MSS focuses on separating the mixture into 4 auditory objects: bass, drums, vocals, and other instruments. The input to the network is the magnitude spectrogram extracted from a music mixture, along with an indicator suggesting which of these 4 objects/sources is of interest. The model predicts the magnitude spectrogram of the object for which the indicator variables is equal to one. The output of the network is compared to the ground truth track and a measure of signal-to-distortion ratio (SDR) is used to evaluate the separation performance.

*Baseline systems*: The performance of the proposed system is compared to a diverse number of other models with comparable setup. Many state of the art systems incorporate specifics about music profiles and extensively optimize to the particular domain. For example, [38, 39] proposes a hybrid model that selects time and/or spectrogram information as suited for the stream. Furthermore, the systems effectively train different networks for each sound class and optimize parameters to consider unique aspects of these classes. For instance, [39] customizes processing based on target sources expected frequency ranges, hence employing a much wider profile for bass sounds relative to vocals. The baseline models used in this study include a single network CNN-based setup [40], a data-augmented deep neural network [41], and a residual network based on de-noising auto-encoder [27] trained on the same data for comparison. All baseline systems have reported competitive performances on music source separation though are slightly below current state-of-the-art systems which rely heavily on a expert knowledge of the characteristics target sources [42].

#### Speech enhancement

To train the speech enhancement model, we use training data from a noisy speech dataset consisting of clean speech data from the Voicebank corpus [43]. These samples are distorted using environmental sounds from the Urban sounds database [44] and the QUT noise set [45] to create a dataset of approximately 30 h of audio at a 16 kHz sampling rate. The noisy speech dataset is created at signal-to-noise ratios (SNR) ranging from − 10 dB to 10 dB to cover a diverse range of low and high SNRs. For testing the model, we use two out-of-training distribution noisy speech datasets: (i) the noisy speech synthetic utterances from the DCASE 2020 challenge [46] consisting of 1500 test examples each of 10-s duration and (ii) a noisy speech database created using the TIMIT speech data [47] corrupted using the BBC sound effects database [48]. A test set of 1000 examples was created using additive noise backgrounds from the Ambience, Animal, Emergency, Office, Technology, Vehicles, and Weather classes in the BBC sound effects database at SNRs ranging from − 10 to 3 dB. Each example contains a single TIMIT speaker, with the examples averaging  27 seconds. In order to extend the evaluation of the system, we train a different instance of the model using the DEMAND dataset [49] consisting of the Voice Banking Corpus with noise recordings from the DEMAND data. This dataset is structured with separate training and testing subsets which affords evaluation of the model using diverse multichannel environmental noises.

In order to address different limitations often associated with performance measure, we employ 3 different metrics in order to evaluate model outcomes from different angles.

*Baseline systems:* The performance of the system for speech enhancement was compared to 3 other baseline neural network architectures evaluated using the same train/test data: (i) a baseline CNN network with the same configuration as that of the proposed system with 5 layers, each consisting 1024 hidden units of size $3 \times 3$ and ReLU activations; (ii) a conventional 5 layer BLSTM (bi-directional long short-term memory), with 1024 hidden units per layer; a feature window of 11 frames of spectral vectors (5 to each side) is used to estimate one output frame; (iii) a baseline generative adversarial network as proposed in [50], with the modified training data used in this work. The baseline models were trained using the Adam optimizer with L1 norm with the clean speech spectrogram as the objective function. These baseline systems are chosen as competitive systems in speech enhancement though precluding others that employ additional factors such as phase information to boost performance [51].

*Ablation evaluations:* By design, the model provides multiple outputs at different points in the network. This structure allows us to evaluate the contribution of different components of the model. In addition, we also perform an ablation study where the contribution of different streams is nullified by setting the embeddings for that stream across all layers to zero. The rest of the model remains unchanged hence allowing us to assess the degree of damage such zeroing has on the final outcome of the model at the output of the stream integrator in layer 2. This analysis is performed on the speech task as example of expected effects of model ablation on overall performance.

## Results

### Music source separation (MSS)

The evaluation of the proposed system is presented in Table 1. The final system performance lists both the combined system $L_{1+2-SI}$ without any feedback as well as the adaptive re-tuning system $TD_{1+2-SI}$ for comparison. The proposed system outperforms the baseline systems for the drums and vocals tracks, while not under-performing on the bass and other categories. The best-performing system with both bass and others track is [41] which uses extensive data augmentation, a very powerful technique to improve generalizability and performance, particularly for recurrent systems.

**Table 1:** Median SDR values for music source separation

Method	Bass	Drums	Others	Vocals
RGT1 [40]	2.70	3.44	2.63	3.84
JY3 [27]	3.67	4.66	3.40	5.74
UHL2 [41]	**5.03**	5.92	**4.19**	5.93
$L_{1+2-SI}$	4.35	5.55	3.69	6.42
$TD_{1+2-SI}$	4.71	**5.95**	3.91	**6.74**

Best performance is marked bold

**Fig. 4:**
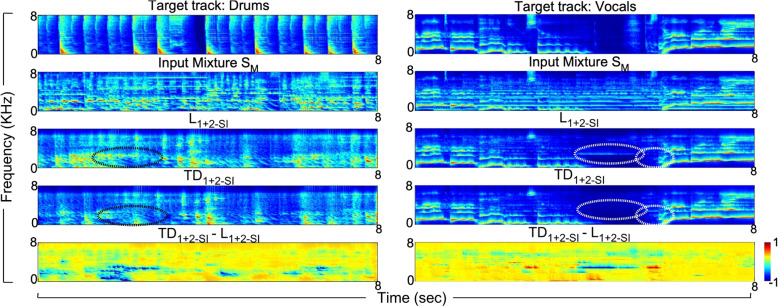
Auditory spectrograms illustrating the workings of the self-feedback mechanism. Row 1:clean spectrogram, row 2:mixture spectrogram, row 3:output of $L_{1+2-SI}$, row 4:output of $TD_{1+2-SI}$, row 5:difference of $TD_{1+2-SI}$ and $L_{1+2-SI}$

To further understand the role of presented self-feedback mechanisms in refining the selection process, we investigate two spectrogram snippets for the drums and vocals (Fig. 4). Rows 1 and 2 show the desired target spectrograms and the input mixture $S_M$ for this specific snippet. Rows 3 and 4 represent the output magnitude spectrograms of systems $L_{1+2-SI}$ and $TD_{1+2-SI}$ respectively, while the spectrogram in row 5 shows the difference of two representations in row 4 and row 3 to highlight the main differences. In both examples, we note that re-tuning plays two important roles: removal of competing tracks that remain in the output and further enhancement of the desired target. In the left column, self-feedback results in the suppression of sustained energy near 0–2 kHz which is prominent in the competing tracks in row 3 (Black circle). Although self-feedback does not completely remove such energy, it strongly suppresses it in row 4, hence improving the representation of the drums by the model. However, this example also highlights that the role of adaptive re-tuning does not bring sufficient temporal precision to better align with the fast dynamics of the specific drum track. In the right column, we observe the absence of strong and sustained energy near 2 kHz in row 4 which is present in the combined system’s output (larges white circle). Hence, self-feedback results in improved matching as compared to the target spectrogram. By the same token, we also observe that self-attention for the same vocals example results in improved representation near the same spectral channels at later time windows (second white circle) further enhancing the vocal activity that needs to be segregated at that moment.

**Fig. 5:**
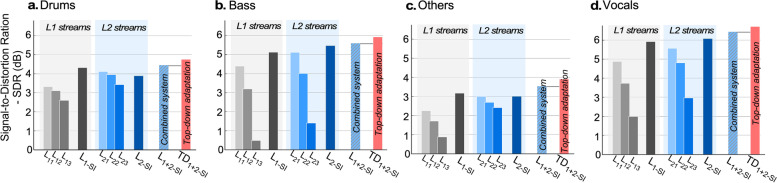
Median signal-to-distortion ratio (SDR) for the MUSDB18 database using the proposed audio separation system. **a**, **b**, **c**, and **d** show the median SDR (in dB) for drums, bass, others, and vocals, respectively. *L*1 streams consist of the parallel paths $L_{11}$, $L_{12}$, $L_{13}$, and stream integrator $L_{1-SI}$. *L*2 streams consist of the parallel paths $L_{21}$, $L_{22}$, $L_{23}$, and stream integrator $L_{2-SI}$. The integrated system $L_{1+2-SI}$ combines the complementary information in both levels 1 and 2 after stream integration and systematically performs better than $L_{1-SI}$ or $L_{2-SI}$. Top-down feedback or self-feedback during inference is shown in $TD_{1+2-SI}$ and shows improvement on all tracks

*Network analysis:* Given the modular nature of the network, it affords a refined analysis akin to an ablation study which allows us to explore the contributions and complementary roles of different components. Median SDR [52] scores of individual streams and stream integrators across levels is presented in Fig. 5 for the music separation task for each of the 4 tracks. The labels ${L_{11},L_{12},L_{13}}$ and ${L_{21}, L_{22}, L_{23}}$ represent the streams in level 1 and level 2, respectively. And label $L_{1-SI}$ and $L_{2-SI}$ represent the performance of the stream integrator in level 1 and level 2, respectively. $L_{1+2-SI}$ represents a combined system, where the output of the stream integrator in each level is added to obtain the signal. The results strongly suggest that individual levels learn complementary information about the attended auditory objects as we ascend the hierarchy, hence supporting our motivation behind combining outputs. A number of observations stand out from these results:

- Among independent streams, faster streams ($L_{11}$ and $L_{21}$) perform best across all tracks, and relatively closely to each other. Comparatively, bass is the only track where the output from the slower stream is significantly weaker than the others. Nevertheless, the stream integrators for all objects (drums, bass, others, vocals) do systematically show an improvement suggesting complementary information gleaned from the 3 streams at each layer of the network.
- The combined system approach $L_{1+2-SI}$ performs better than $L_{1-SI}$ and $L_{2-SI}$ individually, across all 4 soundtracks. Hence, empirically suggesting the presence of complementary information across each level.
- Ultimately, Fig. 5 shows that re-tuning memories during testing results in systematic improvements across all four classes as noted in the results in $TD_{1+2-SI}$.

### Speech enhancement

The same framework is evaluated for speech enhancement but trained on speech data. The first evaluation of the model focuses on the mismatched train/test case, using noisy VoiceCorpus speech training data and DCASE/TIMIT corrupted data (see Methods in Section 3.4.2 for details). Table 2 shows the performance of the speech and noise separation systems in terms of the average PESQ [53], ESTOI [54], and SDR. Both systems $L_{1+2-SI}$ and $TD_{1+2-SI}$, perform better than baseline systems across all metrics. It is also interesting to note that the use of re-tuning leads to a more significant improvement in the SDR measures when compared to intelligibility measures like PESQ.

**Table 2:** Performance in speech denoising

	BBC			DCASE		
Method	PESQ	eSTOI	SDR	PESQ	eSTOI	SDR
BLSTM	1.96	79.77	7.28	1.72	77.92	5.74
CNN	2.08	81.85	7.78	2.15	81.75	6.96
SEGAN	2.02	80.65	6.68	1.99	79.95	6.10
$L_{1+2-SI}$	2.34	84.05	9.20	2.51	83.50	7.92
$TD_{1+2-SI}$	**2.39**	**84.15**	**9.68**	**2.53**	**83.54**	**8.52**

Best performance is marked bold

**Fig. 6:**
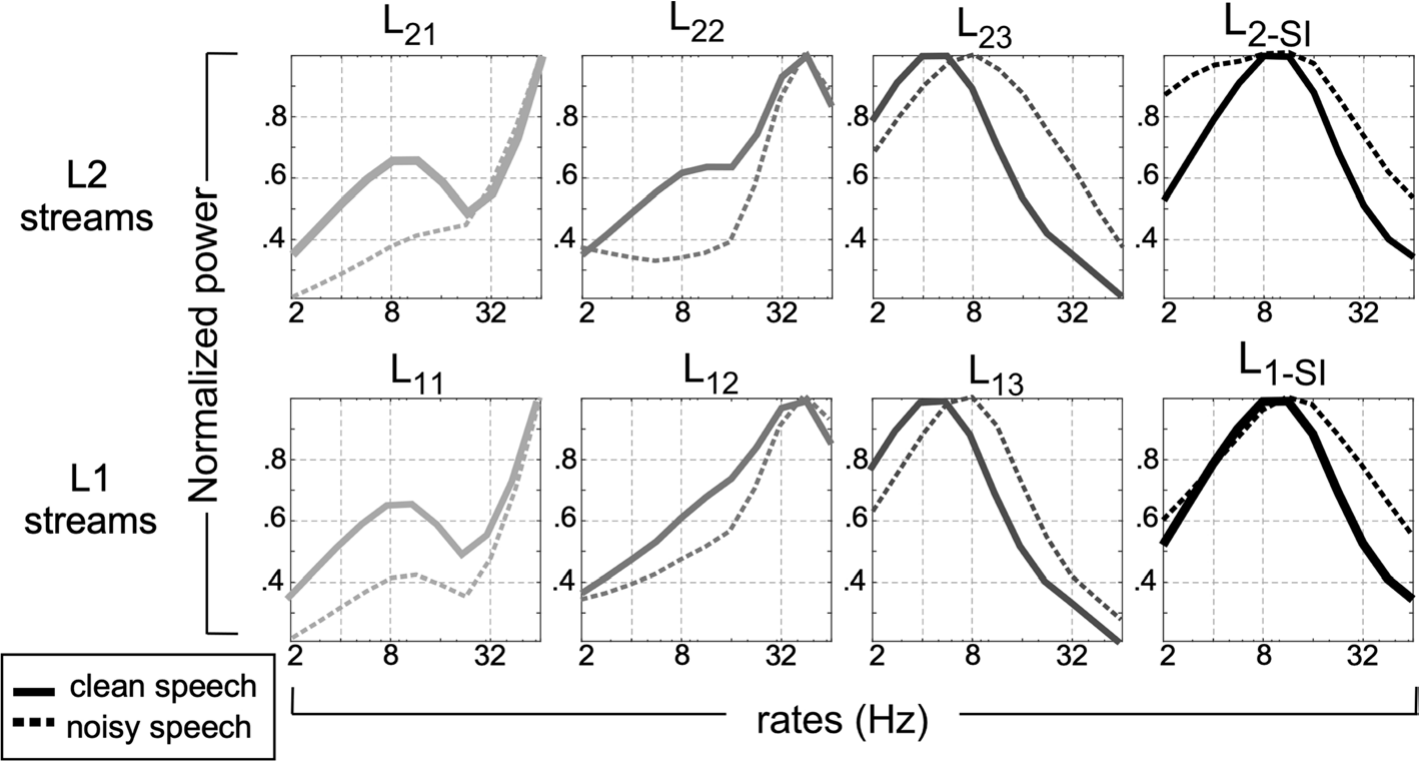
Normalized modulation power spectrum (MPS) averaged across 100 TIMIT utterances, clean (solid line), or with 0 dB white noise (dashed line). The MPS estimate is shown for spectrogram outputs of *L*1 streams (lower row) and *L*2 streams (upper row)

Because of the distributed nature of the processing paths in the system, we can look closely into the mapping profiles that underlie the separability of speech data. We derive the modulation power spectrum (MPS) of an ensemble of 100 randomly sampled speech utterances (from the TIMIT database) processed through the different streams in the model [55–57]. The MPS is an estimate of temporal fluctuations in the signal and reflects how fast sound intensity varies over time. This analysis maps the spectrographic representation of each utterance as viewed by each of the layers in the model into its modulation profile averaged across temporal information to obtain a power density function [58]. Speech utterances with additive white noise at 0 dB are used to examine the changes in spectral profiles between clean and noisy speech. Fig. 6 reveals a comparison of the different modulation power spectra for all streams. The proposed architecture poses the ability to capture different profiles in the modulation spectrum, varying from a high-pass ($L_{i1}$), mid-range ($L_{i2}$), and almost low-pass ($L_{i3}$). The stream integrator $L_{i-SI}$(rightmost column) shows a more typical profile of speech with a peak near 8Hz reflecting the concentration of energy near an average syllabic rate [59].

*Ablation analysis:* To better assess the complementary role of different processing streams, we perform an explicit ablation analysis by nullifying specific streams in the network and evaluating the impact on the final level 2 integrator output. In this evaluation, specific streams (1, 2, or 3) in both layers are set to zero while the rest of the model is evaluated. The effect of leaving one stream out on the system performance as measured by the output of layer 2 is displayed in Table 3 evaluated on the DEMAND train/test dataset (see Methods in Section 3.4.2 for details). The results show a consistent outcome as those noted in the music separation analysis, with complimentary information across the different streams. Specifically, we note that the role of stream 1 and stream 2 may be viewed as somewhat redundant since their ablation results in comparatively similar drops in system performance. The MPS speech profiles in Fig. 6 are consistent with such overlap, while nullifying the third stream does seem to have dramatically affect the performance of the system, in line with the expectation that slower modulation profiles are more critical for fidelity of speech signal [56]. Finally, we also note that the model trained/tested on this new DEMAND dataset -without any ablation- yields speech enhancement results on par with the PESQ values obtained using the evaluation with the TIMIT/DCASE dataset when comparing the last line of Table 3 with values reported in Table 2.

**Table 3:** Speech denoising - ablation studies on DEMAND test set

Stream1	Stream2	Stream3	PESQ
$\times$	$\checkmark$	$\checkmark$	2.08
$\checkmark$	$\times$	$\checkmark$	1.94
$\checkmark$	$\checkmark$	$\times$	1.31
$\checkmark$	$\checkmark$	$\checkmark$	**2.42**

Best performance is marked bold

## Discussion

This study is inspired by the brain’s ability to pick out a target sound amidst other sources that may mask, interfere, or distort the sound of interest. Proposed framework attempts to mimic the following characteristics: (i) distributed processing to facilitate sound segregation, (ii) object memory representing characteristics of sounds across the distributed system, and (iii) selective re-tuning of the target memories to adapt to a particular melody or speaker. Moreover, the proposed system does not incorporate any specific higher-level knowledge or constraints based on the structure of the signal of interest (i.e., semantic or symbolic context). Instead, it is primarily a signal-driven analysis that explores the discriminability of target signals from background distractors based on the distinction between the signal characteristics of classes. Music separation has often benefited from the inclusion of musical syntax and semantic models that incorporate semantic and contextual information [60, 61]. Speech enhancement research has also exploited the highly constrained structure of speech sounds, specifically phonemes [38, 62] which incorporate conditional constraints on the enhancement mappings based on phonemic structure, or even by employing broader contexts imposed by language models [63]. Nevertheless, this machinery of highly specialized processing builds on a common infrastructure that is dealing with the inputs themselves and leveraging constraints in the signal space that can be advantageous for downstream processing. The present study shows that these common, domain-agnostic, principles are a powerful foundation that can set the stage for further improvement in expert systems focusing on speech or music only.

*Distributed multi-scale processing:* The auditory system is viewed as a multi-scale transformation, wherein the spectral and temporal dynamics of an input waveform are extracted across a network of cortical units. Neurons in the auditory cortex are sensitive to spectral energy, spectral modulations, and temporal modulations which capture the rate of change of energy along the temporal axis (rates) in addition to the joint changes in dynamics along time and frequency [12]. Studies have shown that this multi-scale high-dimensional space can separate sound objects from the background, allowing for better target sound recognition [13, 64, 65]. Biology appears to have constrained or optimized this mapping to best represent natural sounds (e.g., speech, nature, animal vocalizations) and acoustic profiles that are constrained by realistic physical attributes (e.g., sounds of musical instruments) [14, 66–68]. Furthermore, recent studies indicate the presence of spatially distributed parallel mappings with rich spectrotemporal space enabling mappings that span different views of the input in addition to a degree of redundancy in the representations [15, 16].

To achieve this rich and multiplexed representation, the proposed system utilizes parallel pathways that are trained independently. Each path is configured to best represent a certain region of the modulation space. As noted in a recent review [69], while the representation is learned in a data-driven way, the chosen configuration of each path is a critical design element of the system and reflects the intended mapping space that we aimed to delineate in each path (Fig. 6). Naturally, an important point to raise here is that the proposed architecture is primarily feedforward with no feedback on the features (except for adaptive retuning). This clearly falls short of the complex interactions and recurrent projections that are present in the auditory cortex and precortical layers [70, 71]. A similar argument is supported by the success of a number of recurrent architectures in source separation tasks [27, 72]. The role of such recurrence is undoubtedly important and has been demonstrated useful in a number of tasks allowing it to capture complex dynamical behaviors and compounded nonlinear functions. Nevertheless, the contribution of such complexity to a system remains ill-understood and does not always guarantee improved behavior and performance without a clear understanding of the role, constraints, and dynamics of the recurrent feedback [73].

*Object memory and temporal coherence:* The role of the network of memories [74, 75] is to act as attentional gates to selectively filter out sounds that are of no interest. This configuration parallels the organic experience of human listeners, where we consciously and deliberately chose to listen to a friend’s voice in a noisy cafeteria, a process referred to as endogenous or top-down selective attention [2, 76, 77]. Unlike uses of the term attention in the context of artificial neural networks and deep learning, the use of attentional gating here is adapted to align with its perceptual meaning, in terms of its role as an information bottleneck that refines inference to align with specific goals [3]. In EMMA framework these object memories are distributed across the parallel paths to leverage the redundant representations in the multi-scale mapping; in line with the distributed nature of memory in the brain which offers a multiplexed view of sensory representations at different levels of granularity and abstractions [18, 19].

Naturally, the current model does not go beyond two layers of the hierarchy and falls far short of the specialized transformations of memory in the brain that span far more than the sensory space and encompass more cognitive regions of the brain [20]. One of the critical processes that these memories leverage in the current system is the principle of temporal coherence. Temporal coherence states that channels that co-vary together tend to be grouped together [17]. This principle is leveraged by the attention mechanism in the brain to bind together channels that are temporally coherent with a target feature [78]. The current work implements this idea using a gating mechanism (Fig. 3) that operates differently from how attention has been used in deep learning applications, effectively as soft search mechanisms using importance weights. This gating uses the memory as an anchor against which temporal coherence is evaluated.

*Selective retuning:* One of the remarkable capacities of the human brain is its ability to adapt to unknown conditions [8, 79]. It is evident from present research in the neuroscience community that there exists a feedback mechanism in the brain that plays a crucial role in how humans navigate unknown environments [80–82]. While the current system is primarily a feed-forward configuration, it relies on a set of priors (object memories) to guide the selection of targets and effectively operate as a self-feedback loop to modulate how incoming signals are processed. In contrast, these priors themselves need not be rigid and should be flexible to reflect specific listening conditions, statistics of the actual target being tracked as well as constraints of the noise conditions at that moment in time.

The adjustments of the priors cannot happen in the design phase of the system, as they have to reflect a specific unknown utterance, melody, or even noise condition. Hence, it is important to develop models that learn continuously. In the current study, we use feedback from stream integrator as guides for this adjustment process; effectively using the model’s output and its posteriors to re-tune its priors. This mechanism can be thought of as a self-correction mechanism that ultimately aims to reinforce the representation of memory with the peculiarities of a specific observation. This approach is also substantiated by a number of findings in the auditory system whereby selective attention is shown to improve the precision of a stimulus already represented in memory [22]. Imposing retuning during inference is limited in the current formulation to adjusting only a few parameters in the model, specifically the object memories.

Overall, this work explores the interaction between feedforward mapping principles and selective modulations (via memories and feedback) to facilitate the segregation of sounds of interest in a complex mixture. Attention in its perceptual interpretation is at the center of this selective adaptation by engaging memories of known objects and adjusting prior knowledge to modulate how incoming sounds are processed. The proposed model is our first attempt at mimicking the auditory system in an end-to-end fashion in a very simple and intuitive setting based on neuroscience studies related to the brain and hearing.

## Data Availability

The datasets used and/or analyzed are open-source and can be found online following the citation. The code base is accessible from the corresponding author on reasonable request.

## Abbreviations

EMMA: Explicit-memory multiresolution adaptive
STFT: Short-time Fourier transform
DCS: Dilated convolution stack
SDR: Signal-to-distortion ratio
MSS: Music source separation
SNR: Signal-to-noise ratio
CNN: Convolutional neural network
PESQ: Perceptual evaluation of speech quality
ESTOI: Extended short-term objective intelligibility
BLSTM: Bi-directional long short-term memory
MPS: Modulation power spectrum
